# Neuro-Modulation for Intractable Pain From Bone Metastasis Using Real-Time Adaptive Motion Management on a Robotic Radiation Therapy Platform: A Case Report and Review of the Literature

**DOI:** 10.7759/cureus.85421

**Published:** 2025-06-05

**Authors:** Seblewongel Enyew, Binbin Wu, Christopher Jackson, Raj Mukherjee, Jordina Rincon Torroella, Daniel Lubelski, Lawrence R Kleinberg, Kristin J Redmond

**Affiliations:** 1 Department of Radiation Oncology and Molecular Radiation, Johns Hopkins University School of Medicine, Baltimore, USA; 2 Department of Neurological Surgery, Johns Hopkins University School of Medicine, Baltimore, USA

**Keywords:** bone metastases, hypophysectomy, neuromodulation, pain, stereotactic radiosurgery

## Abstract

Radiosurgical hypophysectomy has emerged as a promising noninvasive approach for management of intractable cancer pain, particularly in patients with bone metastases, offering a safer alternative to traditional surgical hypophysectomy. Here, we present the case of a premenopausal woman with metastatic breast cancer who developed severe, refractory pain despite conventional pain management. To alleviate worsening pain, the patient underwent radiosurgical hypophysectomy targeting approximately 8 mm of the inferior pituitary stalk and at least 50% of the pituitary gland, using a single 75.0 Gy fraction prescribed to the 50% isodose line. Robotic real-time adaptive radiotherapy was utilized for precision and to avoid unnecessary damage to surrounding critical structures. Following treatment, the patient experienced immediate and significant pain relief. Specifically, pain scores measured using the visual analogue pain score showed a decrease in pain level from 8/10 pre-treatment to 4/10 by day 10 post-treatment. The baseline pain remained around 4-5/10 over time with occasional spikes to 7/10. No meaningful adverse effects were reported, supporting the safety aspect of this approach. These findings are consistent with existing studies that reported immediate pain relief after radiosurgical hypophysectomy, although longer-term outcomes remain variable. This case highlights the potential of radiosurgical hypophysectomy as a valuable option for patients with severe cancer pain, underscoring the need for further clinical studies to validate its effectiveness and explain the underlying mechanisms as well as pain outcome in the long term.

## Introduction

Pain is a frequent and often poorly controlled symptom in individuals with cancer, significantly decreasing their ability to function and overall well-being [1]. According to a meta-analysis study, more than 50% of cancer patients report pain irrespective of their disease stage [1]. Furthermore, a discrepancy has been identified between the manner in which healthcare providers perceive and manage pain compared to how patients and their families experience and manage it [2]. Despite growing recognition, cancer-related pain remains widely undertreated, leading to serious consequences such as impaired function, diminished quality of life, disrupted sleep, and even reduced survival [3,4].

Hypophysectomy has long been studied as a method for controlling intractable cancer pain, particularly in cases involving widespread osseous metastases. It was initially performed as an invasive surgical procedure in the 1950s, and later with chemical ablation approaches. While these early techniques offered significant pain relief, they often resulted in unacceptably high toxicity, including diabetes insipidus, meningitis and death [5-7]. Advances in radiation therapy (RT) technology, including the delivery of precision radiotherapy using frame and frameless systems as well as real-time adaptive motion management on robotic platforms, have revolutionized the potential of neuromodulation for intractable pain from bone metastases. Specifically, this modality offers a safer and noninvasive alternative to earlier surgical techniques with promising efficacy and minimal side effects [8]. Herein, we present a case report of a patient who underwent radiosurgery for neuromodulation for intractable pain from bone metastases, with a marked improvement in pain and quality of life. The case uniquely illustrates the potential of this technique in managing severe, refractory cancer pain, minimizing damage to surrounding tissues, with the intention of raising clinical awareness of this potential technique and encouraging continued investigation.

## Case presentation

A 51-year-old premenopausal woman experiencing pain in her right breast and shoulder presented after palpating a mass in the right breast at the 11 o’clock position. Diagnostic mammography revealed a spiculated lesion characterized by ill-defined margins that persisted on spot compression imaging. Ultrasound-guided core needle biopsy of the mass revealed infiltrating ductal carcinoma that was ER+/PR+/Her-2 negative. The patient was initially treated with breast conservation therapy and adjuvant chemotherapy and then developed a local recurrence and underwent repeat resection and adjuvant RT as well as hormonal therapy. 

**Figure 1:**
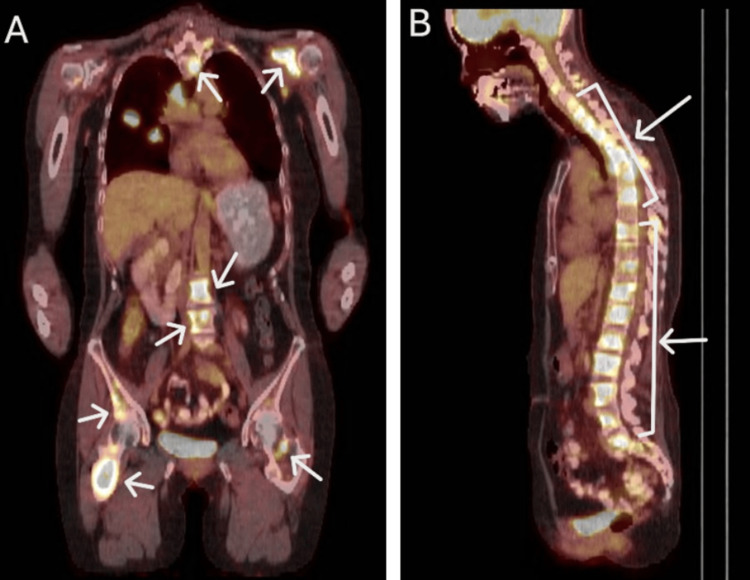
Sagittal (Panel A) and coronal (Panel B) views of positron emission tomography/computed tomography (PET/CT) showing diffusely metastatic disease in the bones, liver and lung.

Approximately nine years later a computed tomography (CT) scan revealed pulmonary lesions, and a biopsy confirmed osseous, hepatic, and pulmonary metastases. Figure 1 shows the positron emission tomography (PET)/CT which revealed diffusely metastatic disease to the bone, liver and lung. Arrows indicate metastatic regions. 

The patient was treated with multiple systemic therapy regimens. In addition, several courses of palliative 3-D conformal RT to sites of painful bone metastases including T3-T7 (30.0 Gy in 10 fractions), C6-T1 (20.0 Gy in five fractions) and the left shoulder (20.0 Gy in five fractions) were conducted. The patient returned to radiation oncology clinic complaining of diffuse osseous pain including in the bilateral scapula, superior spine, bilateral rib (right>left), left shoulder, bilateral hips, and left>right upper humerus. At this time, the patient was taking oxycodone ER, dilaudid, neurontin, Tylenol and dexamethasone, and was drowsy from the regimen but her pain remained 8 out of 10 in intensity on the visual analogue pain scale. The patient had an Eastern Cooperative Oncology Group (ECOG) performance status of 3 (capable of only limited self-care) because of the severity of her pain and was bedridden most of the day. Given the diffuse nature of the pain, there was concern that additional localized RT would be unable to control it. Therefore, radiosurgical hypophysectomy was recommended.

Simulation and radiation treatment planning

The patient was immobilized in an Aquaplast mask in the supine position. She underwent simulation including CT and T1 post-gadolinium MRI both with 1 mm slices. The target was defined as approximately 8 mm of the inferior pituitary stalk (~50%) and at least 50% of the pituitary gland, as visualized on axial and coronal T1-weighted post-gadolinium MRI. Because of the submillimeter accuracy and ability to correct for minor changes in positioning using real-time image guidance on the robotic RT delivery platform, no planning target volume (PTV) expansion was utilized. Critical normal structures including the bilateral cavernous sinuses, optic chiasm, bilateral optic nerves and brainstem were delineated.

**Figure 2:**
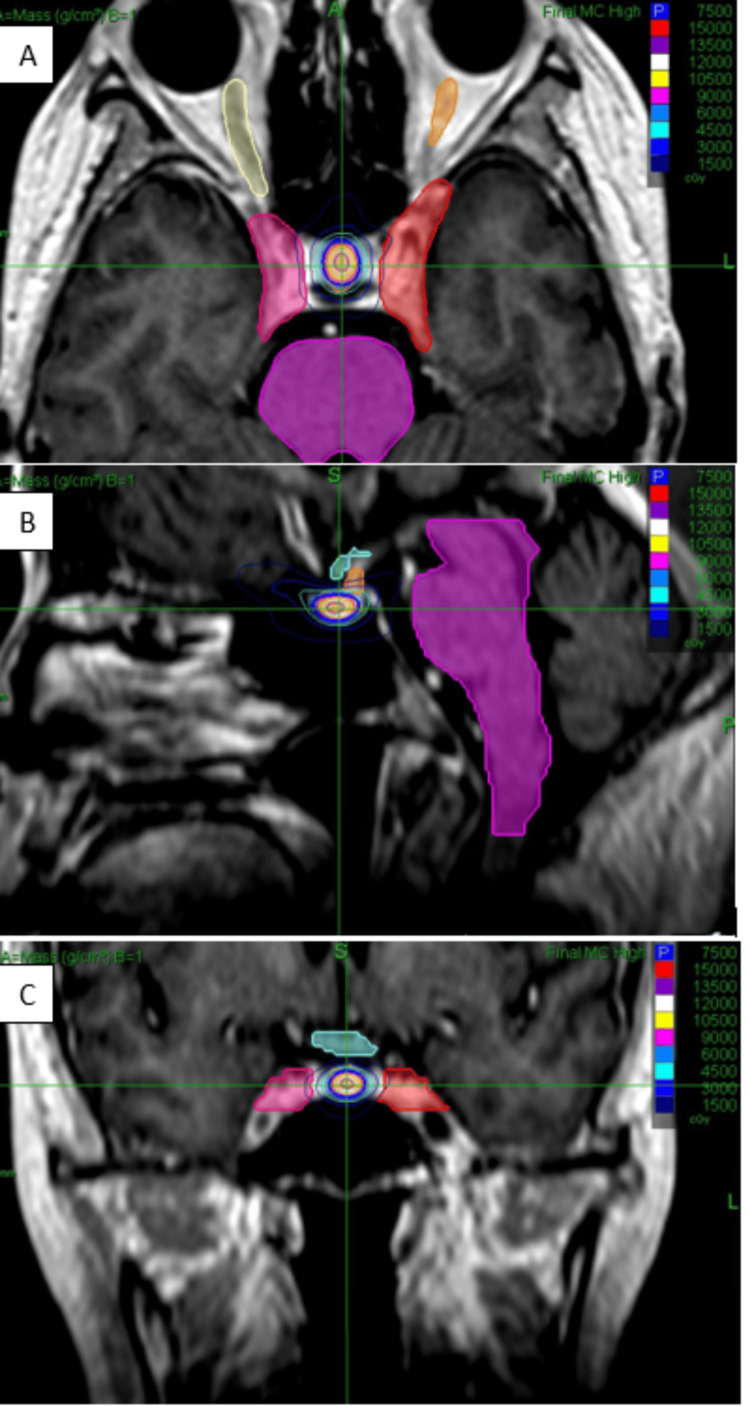
Stereotactic radiosurgery treatment plan and contours (purple region) in axial (Panel A), sagittal (Panel B), and coronal (Panel C) views.

The prescription dose was 75.0 Gy in a single fraction, prescribed to the 50% isodose line. The PTV pituitary coverage was 64.3% with target coverage relaxed as needed in order to achieve normal tissue constraints. The treatment used a fixed collimator type with collimator sizes of 5.0 mm and 7.5 mm. All beams were delivered using a non-isocentric technique. Beam avoidance regions involved the right eye, left eye, and mouth (for beam exit only). The total estimated treatment duration was 71 minutes, with a total monitor unit count of 35,087.5 MU. Figure 2 shows the stereotactic radiosurgery (SRS) treatment plan. Table 1 shows the dosimetry for volumes of interest (VOI).

**Table 1:** Dosimetry table for volumes of interest (VOI) Min = Minimum dose; Max = Maximum dose; Gy = Gray; PTV = Planning target volume

VOI	Volume (cm^3^)	Min (Gy)	Mean (Gy)	Max (Gy)
Optic Chiasm	0.51	1.3	2.8	6.9
Right Eye	9.99	0.2	0.6	2.6
Right Optic Nerve	0.95	0.8	1.9	6.6
Right Lens	0.18	0.3	0.5	0.7
Left Eye	9.66	0.2	0.6	1.4
Left Optic Nerve	0.72	0.8	1.8	7.4
Left Lens	0.16	0.3	0.4	0.7
Brainstem	29.78	0.2	3.0	10.2
Whole Brain	1271.44	0.0	0.7	28.4
Sella	0.53	10.2	66.5	150.0
Infundibulum	0.08	2.1	9.2	29.8
Pituitary	0.26	45.7	96.2	150.0
Left Cochlea	0.18	0.5	0.8	1.4
Right Cochlea	0.13	0.5	1.2	2.1
Right Cavernous Sinus	1.71	1.8	7.6	20.3
Left Cavernous sinus	2.46	1.5	6.2	20.2
PTV_ Pituitary	0.2	5.1	85.3	150.0
Mouth No Beams	196.94	0.0	0.1	1.0

Treatment response

An S-frame with a short mask was used for patient immobilization. The overall treatment time was approximately 71 minutes. During treatment, the patient’s skull positioning tolerance was <10 mm translation (x, y, z axes), < 1° rotation (roll and pitch), and < 3° yaw. The robotic system performed real-time, 6D beam-by-beam offset corrections when the position offsets observed by kV image alignment checks were less than the above positioning tolerance. However, inherent system positioning errors (~0.4 mm), as measured by End-to-End testing, could not be reduced.

At baseline, the patient reported severe pain rated at 8/10 on the visual analogue pain scale. Following stereotactic neuromodulation to the pituitary, there was a significant immediate improvement, with pain levels decreasing to 4/10 by day 10. The pain level persisted at approximately 4-5/10 for the majority of her disease course. However, it is important to note that the patient did experience periodic spikes in her pain most notably at two months post-treatment, when visual analogue pain score rose to 7-10/10 with a strong mechanical component from progressive osseous disease-causing instability. With time this improved and returned to the 4-5/10 level during months four to five, again representing a clinically meaningful improvement from the pre-SRS baseline.

In later follow-ups around six to seven months post-treatment, the patient’s pain levels fluctuated between 2 and 7/10, in part driven by visceral pain from her non-osseous metastatic disease.

The patient’s pain management involved a multimodal approach to address the pain from metastatic breast cancer. At baseline, the regimen included acetaminophen 1000 mg two times daily, gabapentin 300 mg three times daily, HYDROmorphone 4 mg every four hours as needed (PRN), oxycodone ER 15 mg two times daily, and medical marijuana (THC 25 mg, ¼ tab daily PRN). Overall, there were no major increases in opioid or acetaminophen doses in the direct post-treatment period after 10 days. This indicates that the improvement in pain was most likely driven by the SRS rather than changes in medication. The doses of acetaminophen, capecitabine, diazepam, HYDROmorphone, and oxycodone remained unchanged between the 10-day and one-month marks. However, gabapentin was adjusted from three times daily to two times daily, potentially indicating a pain response to the SRS. 

The patient’s pain management then shifted to stronger opioid therapy with morphine ER and PRN hydromorphone being administered at months five and six following SRS. On the other hand, acetaminophen was reduced and gabapentin continued. This likely reflects the multifocal nature of pain in a patient with widely metastatic malignancy. 

In terms of toxicity, the patient did not report any significant toxicity at any point in the disease course following radiosurgical hypophysectomy. Specifically, the patient reported no vision loss or diplopia, and no evidence of pituitary dysfunction, including abnormalities in endocrine dysregulation, was observed throughout the follow-up period. 

The patient ultimately succumbed to metastatic breast cancer at around seven months following SRS.

## Discussion

Stereotactic radiosurgical hypophysectomy represents an optimistic intervention for patients with intractable pain from bone metastases. Although it has been attempted through invasive mechanisms for decades, the toxicity was unacceptably high for a palliative procedure. Advances in radiotherapy, including real-time adaptive motion management on robotic RT platforms, have allowed a resurgence of interest in this topic as it may now be performed non-invasively with data to date suggesting minimal acute or long-term toxicity. In this case, the patient initially reported a severe pain level of 8/10 on the visual analogue pain scale. Immediately following the SRS treatment, there was a significant and sharp decrease in pain level, with pain level decreasing to 4/10 after 10 days. Her pain control was imperfect with episodes in which the pain rose to higher levels. However, her baseline pain remained at approximately 4-5 out of 10 for the majority of the roughly seven months that the patient survived following the treatment, which is undoubtedly a clinically meaningful improvement. In addition, she experienced no adverse effects from the procedure.

We conducted a search on existing literature regarding radiosurgical hypophysectomy for intractable pain from bone metastases. Table 2 summarizes some representative studies we reviewed. 

**Table 2:** Data summary of representative studies regarding radiosurgical hypophysectomy for intractable pain from bone metastases mo = months; NR = not reported; VAS = Visual Analogue Scale, Gy = Gray

Author, Journal, Year Published	Study Type	Number of Patients	Median Follow-up (mo)	Radiation Target	Prescription Dose/number of fractions	Prescription Isodose Line	Pain Response	Toxicity
Hayashi et al., Karger Publishers, 2004 [9]	Prospective	8	6	Border between the pituitary gland and stalk	40 Gy	NR	Six out of 10 were followed up and all showed significant pain reduction	None
May & Liscak, Neuro Endocrinol Lett, 2022 [10]	Retrospective	20	12.6	Pituitary	150-200 Gy	NR	All patients achieved some pain relief. Pain reduction ranged from 0% to 50% of their pre-procedural pain levels.	Hormonal disbalance in three patients, temporary abducens nerve palsy in one patient
Kwon et al., J Korean Neurosurg Soc, 2004 [11]	Prospective	7	7.25	Junction in between neurohypophysis and the pituitary stalk	150-160 Gy	50% isodose covered both the lower part of the pituitary stalk and upper portion of the pituitary gland	Every patient experienced >50% pain reduction in pain. Two patients had worse pain at one month and six months	One patient had transient hypopituitarism and diabetes insipidus
Lovo et al., Cureus, 2019 [12]	Retrospective	11	12.6	Pituitary	150 Gy	50% isodose line	Initially, 80% of patients had reduction of pain from 9 to 3 on VAS scale. End of life pain status showed that 50% had substantial pain, 20% had no effect and 30% died without substantial pain.	Hormonal disbalance in three patients, temporary abducens nerve palsy in one patient

Our findings are consistent with the existing literature, which demonstrates that radiosurgical hypophysectomy leads to significant short-term pain reduction in patients with intractable pain from bone metastases. Previous studies [9,10,12] have reported immediate as well as long-term pain relief after this procedure. Similarly, our patient also experienced a significant drop in pain levels shortly after treatment. However, longer-term outcomes turned out to be more variable. For instance, one study noted a return of pain in two out of seven patients at one and six months post-treatment [11], which is consistent with our findings. Our patient initially had reduced pain closely after the procedure, but pain levels began to rise again around two months post-treatment, followed by further fluctuations at six to seven months. 

The mechanism for early pain relief following radiosurgical hypophysectomy may be due to an increase in cortisol levels following treatment. Takeda and colleagues demonstrated that after chemical neurolysis of the pituitary, cerebrospinal fluid (CSF) levels of adrenocorticotropic hormone (ACTH) significantly increased both shortly after the surgery and for two months post-hypophysectomy [13]. This is consistent with the patient reported herein, in which her pain levels improved rapidly in the days following treatment. More importantly, the study found that patients who did not exhibit this increase in CSF ACTH did not have a significant reduction in pain. ACTH is known to be a hormone with anti-inflammatory and analgesic effects via stimulation of the adrenal glands to release cortisol [13]. 

Although the patient experienced a surge in pain around two months after treatment, overall pain levels subsequently improved, stabilizing within a range of 4 to 5/10 by the fourth and fifth months. This decrease in pain in later follow-ups may be explained by a recently proposed framework in a historical review article [14]. After examining various studies, the review put forward a hypothesis involving the role of the hypothalamic-pituitary axis. Specifically, radiosurgery may promote the redistribution of oxytocin and vasopressin hormones via hypothalamic-pituitary modulation, resulting in immediate as well as long-term pain relief [14]. This new insight is based on accumulating evidence that suggests that analgesia from radiosurgery may result from radio-endocrine modulation of the hypothalamus, rather than from direct pituitary destruction [9,15,16]. It proposes that radiation may partially disrupt the pituitary stalk, causing hypothalamic hormones, such as oxytocin and vasopressin, to be released into the CSF, where they modulate central pain-inhibitory pathways.

The hypothesis mirrors the second phase of triphasic diabetes insipidus, where pituitary stalk injury causes hypothalamic hormones like vasopressin and oxytocin to leak into the CSF, activating central pain-inhibiting pathways [14]. Even though oxytocin was found to have a short half-life, it’s known to reduce pain by affecting pain pathways [17]. Vasopressin, also involved in pain modulation, increases in the CSF after surgery [14,17]. The rise in these hormones after radiosurgery hypophysectomy might explain the immediate pain relief seen in the patient.

Lastly, the hypothesis offers that as the acute hormonal increase in oxytocin and vasopressin declines, regenerative changes in the hypothalamic-pituitary axis lead to longer-term pain relief [14]. This postulation is based on animal and human studies that have shown regeneration of the hypothalamus and pituitary. According to the review, these studies observed recovery as early as three weeks in posterior pituitary of preclinical models and as much as two years in hypothalamic nuclei in humans [14]. Collectively, these observations suggest a potential dual-phase model by which hypophysectomy relieves pain. This involves hormone-mediated analgesic response due to leakage into the CSF followed by long-term pain relief due to hypothalamic regeneration.

The spike in pain level was observed two months after radiosurgery, and fluctuations are anticipated in patients with widely metastatic cancer from a variety of causes. For example, osseous cancer pain is often caused by instability of involved bones [18]. Since radiosurgical hypophysectomy would not stabilize the bones, it would not be expected to reduce mechanical pain. Similarly, cancer patients frequently experience pain from involvement of visceral organs which would not be managed through radiosurgical hypophysectomy. Metastases to these regions are known to cause significant nociceptive and neuropathic pain through various mechanisms such as nerve compression, inflammation, and organ dysfunction [18]. As such, the occasional variability in pain levels likely does not represent failure of the procedure. Multiple confounding factors, including disease progression and organ-specific complications, likely contributed to the patient’s overall pain experience, making it difficult to isolate the effect of the radiosurgery.

Overall, this case report supports the growing body of literature that stereotactic radiosurgery to the pituitary region may offer meaningful pain mitigation in patients, particularly those with hormonally mediated metastatic disease affecting the neuroendocrine system. The findings contribute to existing literature and illustrate the potential therapeutic application of this treatment in palliative care.

This case report is limited by its sample size as it presents data from a single patient. Larger-scale retrospective and prospective studies will be critical to validate these findings and the mechanisms of pain relief following this treatment. Additionally, we reported a patient with a hormone-responsive tumor. As a result, this raises the possibility that such outcomes may be more exclusive to patients with hormonally mediated malignancies. Future studies should investigate the role of hormonal responsiveness in pain management and treatment outcomes.

## Conclusions

This case report illustrates the potential of stereotactic radiosurgical hypophysectomy as a procedure to manage intractable cancer pain with minimal invasiveness and toxicity. While the patient demonstrated a significant reduction in pain level shortly after the treatment, her pain levels remained variable over time, likely reflecting the multifocal nature of pain in patients with widely metastatic disease. Given the encouraging initial outcomes and the plausible mechanisms involving hormone-mediated analgesia, further studies are necessary to verify the underlying mechanisms, identify potential confounding variables, and evaluate criteria for long-term efficacy. Ultimately, a more comprehensive understanding of this technique could broaden its application in managing refractory cancer pain. 
